# Vocal Fatigue als Indikator für komplexe Stimmstörungen – eine diagnostische und therapeutische Herausforderung

**DOI:** 10.1007/s00106-022-01186-6

**Published:** 2022-07-04

**Authors:** L. E. Stappenbeck, S. Bartel, M. Brockmann-Bauser

**Affiliations:** 1 Abtl. für Phoniatrie und Pädaudiologie, Universitätsklinik und Poliklinik für Hals-Nasen-Ohren-Heilkunde, Kopf- und Hals-Chirurgie, Hallesches Hör- und ImplantCentrum, Universitätsklinikum Halle (Saale), Ernst-Grube-Str. 40, 06120 Halle (Saale), Deutschland; 2 Abteilung Phoniatrie und Klinische Logopädie, Klinik für Ohren‑, Nasen‑, Hals- und Gesichtschirurgie, Universitätsspital Zürich, Zürich, Schweiz; 3 Universität Zürich, Zürich, Schweiz

**Keywords:** Vocal Fatigue Index (VFI-D), Stimmstörungen, Vocal Fatigue Index (VFI-D), Muscle tension dysphonia

## Abstract

Eine alltagseinschränkende, pathologische Stimmermüdung (Vocal Fatigue) wird v. a. bei BerufssprecherInnen (z. B. LehrerInnen) zunehmend beobachtet und als Indikator für komplexe Stimmstörungen betrachtet. Da bislang wenig systematische Studien zu diesem Phänomen existieren, fehlt bislang eine einheitliche Diagnostik. Anhand der Kasuistik eines Berufssprechers werden Möglichkeiten einer strukturierten Erhebung von Vocal Fatigue vorgestellt und im Hinblick auf ihre laryngoskopischen, instrumentell akustischen („performance fatigue“) und subjektiven Merkmale („perceived fatigue“) diskutiert. Zudem wird eine Abgrenzung zu den Modellen Vocal Effort, Vocal Demand und Vocal Demand Response nach Hunter et al. (2020) vorgenommen.

## Abstract (en)

Vocal fatigue is increasingly observed among professional voice users (e.g., teachers). This pathologic state is associated with restrictions in everyday life and is considered to be an indicator of complex voice disorders. Since there are few systematic studies on the phenomenon, there is currently no uniform definition or diagnostic workup. Based on the case study of a professional speaker, possibilities for a structured vocal fatigue assessment are presented. These are discussed with regard to their laryngoscopic, instrumental acoustic (“performance fatigue”) and subjective symptom-related characteristics (“perceived fatigue”). In addition, a differentiation from the vocal effort, vocal demand, and vocal demand response models according to Hunter et al. (2020) is made.

Stimmermüdung kann bei Stimmgesunden als normale Reaktion auf eine erhöhte Stimmanforderung auftreten [6]. Eine alltagseinschränkende, pathologische Stimmermüdung (Vocal Fatigue) muss hiervon abgegrenzt werden [12, 14, 15]. In dieser Arbeit soll das zunehmend auftretende Phänomen der Vocal Fatigue nach aktuellen Gesichtspunkten vorgestellt und deren diagnostische Fallstricke anhand der Kasuistik eines Berufssprechers erläutert werden.

## Falldarstellung

### Anamnese

Schätzungen zufolge sind bis zu 33 % aller Erwachsenen und ca. 41 % der „professional voice users“ (BerufssprecherInnen) in ihrem Berufsleben von Stimmstörungen betroffen. „Professional voice users“ weisen im Vergleich zu anderen Subgruppen doppelt so häufig funktionelle Stimmbeschwerden auf wie andere Gruppen [10, 29]. Vocal Fatigue (VF) wird im Zusammenhang mit funktionellen Stimmstörungen diskutiert [20]. Dieses Phänomen scheint jedoch in gewissem Maße von Komorbiditäten (z. B. Reflux, Allergie, Depression) beeinflusst [1], wodurch es wahrscheinlich auch unabhängig auftreten kann [26, 31].

Obwohl Vocal Fatigue in der Literatur implizit als wiederkehrende, kontinuierlich steigende Stimmanstrengung bei Stimmgebrauch und Verbesserung dieser bei stimmlicher, körperlicher und kognitiver Entspannung verstanden wird, fehlt bisher eine einheitliche Diagnostik [14, 15, 26, 31] Es besteht jedoch ein allgemein anerkannter Konsens dazu, dass Vocal Fatigue anhand der Merkmale „performance fatigue“ (messbare stimmliche Leistungseinschränkung) und „perceived fatigue“ (subjektive Wahrnehmung von alltagseinschränkender Stimmermüdung) beschrieben wird [14, 15]. Da die Studienlage aktuell noch heterogen ist, wird Vocal Fatigue vorrangig anhand ihrer subjektiven Merkmale, z. B. über den Fragebogen Vocal Fatigue Index (VFI), diagnostiziert [21, 26, 31].

Für VF wird ein multifaktorielles Modell angenommen, welches subjektiv empfundene Stimmbeschwerden sowie mögliche physiologische Defizite umfasst. Gemäß Hunter et al. (2020) sollte VF von Vocal Effort, Vocal Demand und Vocal Demand Response abgegrenzt werden (Tab. 1; [14, 15]). Besonders BerufssprecherInnen (z. B. LehrerInnen) können in der Ausübung ihrer Tätigkeit massiv beeinträchtigt sein, wenn Häufigkeit und Schweregrad von VF zunehmen [2, 3, 13, 18].

**Table 1:** 

Begriff	Definition
*Beschreibungen von Stimmermüdungssymptomen nach Imhofer (1913) und Nawka/Wirth (2008) *[17, 22]	
Reasthenie und Phonasthenie	Konzept der abnormen Ermüdung der Sprech- und/oder Singstimme, welches seit dem 19 Jh. im deutschsprachigen Raum beschrieben ist. Dieses ist als konstitutionelle Schwäche des Stimmapparats ohne Heiserkeit oder objektivierbare laryngostroboskopische Symptomatik definiert und resultiert in einer stimmlichen Fehl‑/Überlastung mit evtl. akustischen bzw. physiologischen Konsequenzen. Eine Integration bzw. Abgrenzung zum Konzept der Vocal Fatigue ist bislang nicht erfolgt
*Beschreibungen von Stimmermüdungssymptomen nach Solomon (2008) *[26]	
Vocal Load/Vocal Loading	Stimmfordernde Aufgaben, die im Belastungsprozess (Vocal Loading) zu subjektiven Missempfindungen, erhöhter Muskelspannung im Hals-Nacken-Bereich, verstärkter Taschenfaltenaktivität sowie erhöhtem subglottischem Druck und gesteigerter Sprechstimmlage führen können. Umfang und Ausprägung der Symptome beschreibt die situationsspezifische Stimmlast (Vocal Load)
*Aktueller Konsens zur Beschreibung von Stimmermüdungssymptomen nach Hunter et al. (2020) *[14]	
Vocal Demand	Stimmanforderung, welche sich aus einer Kommunikationssituation ergibt. Diese ist unabhängig von der Physiologie, Stimmtechnik oder Situationswahrnehmung des/der SprecherIn
Vocal Demand Response	Die individuelle Art des/der SprecherIn, auf ein Vocal Demand zu reagieren. Sie beinhaltet sowohl subjektiv, als auch objektiv messbare Parameter
Vocal Effort	Die subjektiv wahrgenommene stimmliche Anstrengung des/der SprecherIn während eines Vocal Demand, welcher auch vom Hörer wahrgenommen werden kann. Dieser wird über subjektive Fragebögen und beispielsweise das Item „S“ der GRBAS-Skala erhoben (GRBAS = „grade“ [Grad der Heiserkeit], „rough“ [Rauigkeit], „breathy“ [Behauchtheit], „astenic“ [Verlust an Klangfülle], „strain“ [Anstrengung – gepresst])
Vocal Fatigue	Ein multifaktorielles Konzept, welches die subjektive Wahrnehmung der eigenen stimmlichen Leistungsfähigkeit und/oder physiologische stimmliche Defizite integriert und sich symptomatisch durch einen erhöhten Vocal Effort oder neuromuskuläres Defizit äußern kann. Bislang ist nicht abschließend geklärt, welche möglichen Einflussfaktoren Vocal Fatigue bedingen

Während der zweiten Welle der Corona-Pandemie stellte sich ein selbstständiger Gesangslehrer im Alter von 64 Jahren („professional voice use level II“ [29]) ohne bekannte Vorerkrankungen zur phoniatrischen Diagnostik vor. Er beschrieb eine steigende Stimmanstrengung, die immer früher während des Unterrichtens einsetze sowie Schmerzen auf Höhe des Jugulum. Die Beschwerden bestünden, seitdem der Patient trotz einer akuten Erkältung mit Heiserkeit eine berufsbegleitende Weiterbildung zum Chorleiter inkl. Gesangsausbildung durchlaufen habe.

Somit lagen während einer stimmlichen Leistungseinschränkung sowohl eine hohe Stimmbelastung im Rahmen der Weiterbildung, aber auch beruflich anspruchsvolle Tätigkeiten im Rahmen der Corona-Pandemie vor.

### Diagnostik und klinische Befundung

Aktuell besteht kein Konsens, welche konkreten Untersuchungen zur VF in den Merkmalen „performance fatigue“ und „perceived fatigue“ erfolgen sollen. Folgende Untersuchungen sind in der bisherigen Literatur u. a. angewendet worden:

- „*perceived fatigue*“: Erfassung der subjektiven Wahrnehmung von alltagseinschränkender Stimmermüdung über Fragebogenerhebungen [3, 14, 15, 21, 26, 31]
- „*performance fatigue*“: Beurteilung der Stimmlippenschwingung und des Glottisschlusses [6, 9, 16]; Beschreibung verschiedener aerodynamischer Parameter [2, 7, 12]

Des Weiteren scheinen weitere Faktoren einen Einfluss auf die Entwicklung und Aufrechterhaltung von VF zu haben:

- subjektives Stresserleben der Betroffenen [5, 17, 24]
- laryngeale Entzündungsgeschehen in der (prä)diagnostischen Phase, die auf die Präsenz von Mikrowunden hindeuten könnten [23, 28]
- Fehlspannungen v. a. der extrinsischen Muskulatur des Hals-Nacken-Bereichs aufgrund einer konstitutionellen Schwäche der intrinsischen Larynxmuskulatur z. B. durch Fehl‑/Überlastung [7, 30]

Für diese Kasuistik wurde eine standardisierte Stimmdiagnostik gemäß Protokoll der European Laryngological Society (ELS) mit multiprofessioneller Differenzialdiagnostik durchgeführt [11].

#### Visuelle laryngoskopische Befundung

Laryngoskopisch zeigten sich die Stimmlippen beidseits leicht gerötet mit Schleimauflagerungen. Es bestand spontan ein ovalärer Restspalt in Phonationsstellung, wobei ein kompletter Stimmlippenschluss mit regelgerechter Motilität möglich war. Zusätzlich bestand eine leichte Hyoidasymmetrie nach rechts. Die Histologie zeigte eine plattenepitheliale Schleimhaut mit Akanthopapillomatose und Parakeratose neben einer geringgradig chronischen Entzündung mit mäßiggradiger Schleimhautfibrose. Es bestand kein Anhalt für Malignität.

#### Stimmdiagnostik (perzeptive und instrumentelle akustische Untersuchung)

**Figure 1:**
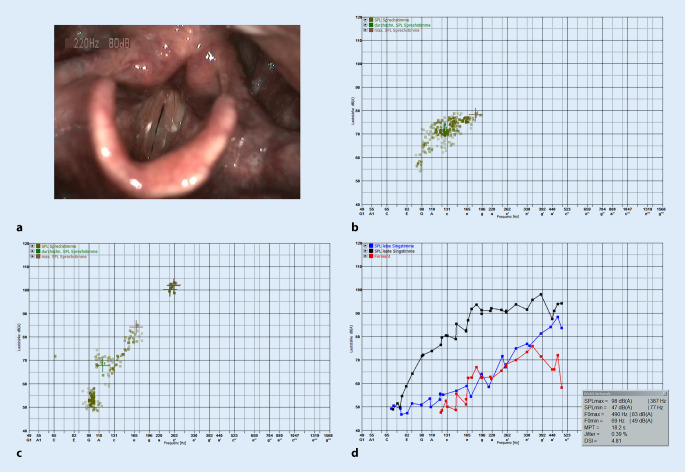

Perzeptiv wurde die Stimme als wechselnd leicht heiser und klar bei inkonstantem Räusperzwang eingeschätzt (R1B1H1). Die Stimmeinsätze waren teilweise knarrend und der Stimmansatz eher rückverlagert. Spontan lag die mittlere Sprechstimmlage (MSSL) zwischen 123–130 Hz, in der Prüfsituation jedoch tiefer auf 92,5 Hz. Alle weiteren akustisch erhobenen stimmlichen Leistungsparameter inkl. des Stimmbelastungstestes nach Seidner [25] entsprachen der Norm (Modulationsbreite: 15 Halbtöne (HT), Tonhaltedauer: 18 s, max. Lautstärke: 104 dB). Auch der Singstimmklag war teilweise knarrend/belegt, wobei eine ausgewogene Resonanz und Klangfarbe bei Anwendung der Singtechnik möglich war. Der Singstimmumfang war mit 35 HT und mit einer Dynamikbreite von 51 dB regelgerecht. Auffallend war eine abgesunkene Sängerformantenkurve (Abb. 1). Der Dysphonia Severity Index (DSI) lag bei 4,81, was als nicht dysphon gewertet wird.

#### Elektroglottographie

Bei normal lauter Phonation ergab sich ein Quasi-Geschlossen-Quotient (CQ) zwischen 0,40 und 0,42, was als noch regelgerecht betrachtet wird. Bei lauter Phonation ergab sich ein CQ zwischen 0,63 und 0,64, was als regelgerecht betrachtet wird.

#### Subjektive Beschwerden

Im Voice Handicap Index 9 international (VHi9i) [8] wurde ein Gesamtscore von ∑ = 17 (von insgesamt 40) erreicht, wobei physische Symptome die meisten Punkt erhielten (Unterscores: „funktional“: ∑ = 4 von 16; „physisch“: ∑ = 10 von 12; „emotional“: ∑ = 1 von 8). In der Vocal Tract Discomfort Scale (VTD) [19] wurden die Empfindungen „Trockenheit, Schmerzen, Reizung und/oder Kloß im Hals“ in Häufigkeit und Schweregrad als sehr oft bis immer (Skala 4–6 von 6) angegeben. Im Vocal Fatigue Index (VFI-D) [4, 27] wurde ein Gesamtscore von ∑ = 60 von 76 ermittelt, wobei die Unterscores „Fragen zu Beschwerden im Hals-Nacken-Bereich“ mit ∑ = 20 von 20 und „Fragen zur Verbesserung der subjektiven Symptomatik nach allgemeinem Ausruhen“ mit ∑ = 11 von 12 am höchsten bewertet wurden. Die Auswertung der Fragebögen deutete auf eine Stimmstörung mit Missempfindungen und Fehlspannungen im Hals-Nacken-Bereich sowie ausgeprägter VF-Symptomatik hin.

#### Weiterführende Differenzialdiagnostik

In der psychologischen Beurteilung wurde kein voll ausgeprägtes depressives Syndrom festgestellt. Es wurde jedoch festgehalten, dass zum Diagnosezeitpunkt der Patient einen massiven Leistungsanspruch an sich selbst zeigte. Es fehlten in Anbetracht der bestehenden berufseinschränkenden Stimmstörung effiziente Entspannungs- und Stressbewältigungsmechanismen, weshalb eine begleitende psychotherapeutische Behandlung empfohlen wurde.

Aufgrund anhaltender Schmerzen im Hals-Nacken-Bereich sowie zusätzlich auftretender subjektiver Schluckbeschwerden und Schwächegefühle in den Armen wurde eine Magnetresonanztomographie (MRT) der Halswirbelsäule durchgeführt. Es zeigte sich als Hauptbefund eine aktivierte Spondyloosteochondrose mit begleitender Unkarthrose im Bewegungssegment der Halswirbelkörper (HWK) C5–6.

Folgende Faktoren werden für die vorliegende Kasuistik als begünstigend für eine funktionelle Stimmstörung und das Phänomen der Vocal Fatigue angenommen:

- *organisch*: bestehende geringgradige chronische Laryngitis mit Kompensation durch die extrinsische Larynxmuskulatur bei ausgebildeter Sprech- und Gesangsstimme
- *konstitutionell*: Hyoidasymmetrie, degenerative Prozesse in HWK-Bewegungssegmenten
- *habituell*: v. a. ineffizienter Einsatz der Sprech- und Gesangsstimme als Folge einer kompensatorischen Fehl- bzw. Überlastung im Rahmen einer Laryngitis bei gleichzeitiger Gesangsweiterbildung
- *ponogen*: hohe berufliche Stimmbelastung bei verringerter ganzkörperlicher Aktivität im Rahmen des Teleunterrichts während der Corona-Pandemie
- *psychogen*: hoher Selbstanspruch bei zum Erkrankungszeitpunkt nicht ausreichenden Entspannungs- und Stressbewältigungsmechanismen

Es zeigten sich bei dem Patienten somit Merkmale einer Hyperfunktion (z. B. im Sinne einer Muscle Tension Dysphonia) und auch Dysodie. Zudem konnten degenerative muskuloskeletale und psychologische Komponenten nicht ausgeschlossen werden. Ein Vorschlag für die zusammenfassende Beurteilung für diesen Patienten ist: *funktionelle Dysphonie und Dysodie bei Hyperfunktion der extrinsischen Larynxmuskulatur nach grippalem Infekt mit Laryngitis (mit vermutlich zervikogener Beteiligung); Leitsymptomatik Vocal Fatigue bei psychosomatischer Mitbegünstigung.*

## Diskussion

Die vorgestellte Kasuistik zeigt auf, dass „professional voice users“ wie BerufssprecherInnen (z. B. GesangslehrerInnen), welche das Phänomen Vocal Fatigue als Leitsymptom beschreiben, komplexe Stimmstörungen aufweisen können. Initial können dabei Merkmale der „performance fatigue“ überwiegend unauffällig sein.

VF ist demnach in dieser Kasuistik als Symptom einer Stimmstörung zu betrachten, das in der phoniatrischen Standarddiagnostik aktuell nur über die Merkmale der „perceived fatigue“, z. B. Fragebogenerhebungen, effizient ermittelt werden kann. Da es jedoch laut allgemeinem Konsens auch unabhängig von Stimmstörungen auftreten kann, muss es prinzipiell als multidimensionales Phänomen gewertet werden.

Nach aktuellem Konsens muss Vocal Fatigue von anderen Phänomenen der stimmlichen Ermüdung abgegrenzt werden (Tab. 1). Zudem scheint eine Vielzahl bedingender Faktoren zu existieren, welche zum jetzigen Zeitpunkt noch nicht systematisch beschrieben sind. Daher scheint neben einer multiprofessionellen Diagnostik auch ein ganzheitlicher therapeutischer Ansatz inklusive Stressmanagement-Angebot angemessen [29].

Weiterführende Studien sollten das Ziel verfolgen, einen einheitlichen Standard in der Diagnostik von VF zu erarbeiten. Nur so können die therapeutischen Möglichkeiten optimiert werden, um v. a. BerufssprecherInnen eine rasche Wiederaufnahme ihrer Arbeitstätigkeiten zu gewährleisten.

In der Literatur werden mehrere Begriffe zur Beschreibung von Vocal Fatigue herangezogen (Tab. 1).

## Fazit für die Praxis

Vocal Fatigue …

- ist nur über einen multiprofessionellen Ansatz zu diagnostizieren und zu therapieren.
- kann im Merkmal der „perceived fatigue“ effizient über den Vocal Fatigue Index (z. B. VFI-D) systematisch erhoben werden.
- ist ein multidimensionales Phänomen, das von den Modellen Vocal Effort, Vocal Demand und Vocal Demand Response differenziert werden sollte.
- bedarf weiterführender systematischer Forschung zur Erarbeitung effektiver Therapieansätze.
